# Vibrational Properties and DFT Calculations of Perovskite-Type Methylhydrazinium Manganese Hypophosphite

**DOI:** 10.3390/molecules25215215

**Published:** 2020-11-09

**Authors:** Aneta Ciupa-Litwa, Maciej Ptak, Edyta Kucharska, Jerzy Hanuza, Mirosław Mączka

**Affiliations:** 1 Institute of Low Temperature and Structure Research, Polish Academy of Sciences, 50-422 Wrocław, Poland; a.ciupa@intibs.pl (A.C.-L.); j.hanuza@intibs.pl (J.H.); m.maczka@intibs.pl (M.M.); 2 Department of Bioorganic Chemistry, Faculty of Production Engineering, University of Economics and Business, 118/120 Komandorska str., 53-345 Wrocław, Poland; edyta.kucharska@ue.wroc.pl

**Keywords:** hypophosphite, methylhydrazinium cation, DFT, Raman, IR

## Abstract

Recently discovered hybrid perovskites based on hypophosphite ligands are a promising class of compounds exhibiting unusual structural properties and providing opportunities for construction of novel functional materials. Here, we report for the first time the detailed studies of phonon properties of manganese hypophosphite templated with methylhydrazinium cations ([CH_3_NH_2_NH_2_][Mn(H_2_PO_2_)_3_]). Its room temperature vibrational spectra were recorded for both polycrystalline sample and a single crystal. The proposed assignment based on Density Functional Theory (DFT) calculations of the observed vibrational modes is also presented. It is worth noting this is first report on polarized Raman measurements in this class of hybrid perovskites.

## 1. Introduction

In recent years, a lot of attention has been devoted to hybrid organic-inorganic compounds of general formula ABX_3_ (A = organic cation; B = divalent metal cation; X = halide, formate (HCOO^−^), azide (N_3_^−^), cyanide (CN^−^), dicyanamide (N(CN)_2_^−^, dca*^−^*), hypophosphite (H_2_PO_2_^−^)), crystallizing in a perovskite-type architecture due to their interesting physicochemical properties including photovoltaic [1], light-emitting [2,3,4,5], multiferroic [6,7,8], barocaloric [9,10], and non-linear optical (NLO) [11,12,13] properties. The structure-related properties of these compounds depend on flexibility of ligands, size and shape of organic cations as well as their ability to form hydrogen bonds (HBs) with the anionic framework. In this respect, very large flexibility of the dca^-^ ligands and pronounced temperature-induced disorder of organic cations at elevated temperatures is responsible for unusual softness and promising barocaloric properties of dicyanamides [9,10,14,15]. Order-disorder phenomena are also responsible for onset of spontaneous polarization in formates and their multiferroic properties [6,8]. These phenomena can be tuned by proper use of organic cations and we showed for the first time in 2017 that methylhydrazinium cation (CH_3_NH_2_NH_2_^+^, MHy^+^) may also be used for the construction of perovskite-type formates [8]. Very recently, we showed that MHy^+^ also allows extending the formation of three-dimensional (3D) lead halides beyond two already known methylammonium and formamidinium analogues [12,13]. This cation also allows constructing the two-dimensional (2D) perovskite of the formula MHy_2_PbI_4_ [5] as well as 3D hypophosphite [MHy][Mn(H_2_PO_2_)_3_] [5].

While perovskite-type halides, formates, azides, cyanides, and dicyanamides have been known for a long time, their hypophosphite analogues were discovered for the first time in 2017 [16]. Except for one magnesium analogue [17], all known compounds contain manganese as divalent cation [5,16,18,19]. Literature data show that although H_2_POO^−^ ligand has a very similar size to the formate ion, the hypophosphite frameworks show much higher flexibility than their formate counterparts [16,19]. As a result, hypophosphites have higher potential than formates for structural distortion, which results in the presence of unconventional tilts of MnO_6_ octahedra and columnar shifts [16,19]. Furthermore, they often show pronounced off-centering of organic cations away from the cavity center [16,19].

Up until now, Raman and IR studies were reported for only one hypophosphite, i.e., formamidinium manganese hypophosphite [20]. Regarding methylhydrazinium compounds, we reported Raman and IR data for some formates and two halides (MHy_2_PbI_4_ and MHyPbBr_3_). However, deep understanding of vibrational modes of MHy^+^ cations is still lacking and vibrational properties of recently discovered [MHy][Mn(H_2_PO_2_)_3_] are not known. We decided, therefore, to analyze vibrational properties of [MHy][Mn(H_2_PO_2_)_3_] crystal and propose reliable assignment of vibrational bands by performing Density Functional Theory (DFT) calculations.

## 2. Experimental Section

### 2.1. Synthesis of Single Crystals

[MHy][Mn(H_2_POO)_3_] crystals were grown by slow evaporation at 50°C of solution prepared by dissolving 5 mmol of MnCO_3_ (99.9%, Sigma-Aldrich, Saint Louis, MO, USA) and 10 mmol (0.53 mL) of methylhydrazine (98%, Sigma-Aldrich) in 6.46 mL of aqueous solution of hypophosphorous acid (50%, Sigma-Aldrich) (equivalent of 60 mmol of H_3_PO_2_). Further details can be found in our previous paper [5].

### 2.2. Materials and Methods

Polycrystalline IR spectra were measured using a Nicolet iS50 FT-IR spectrometer. The standard KBr pellet method was used for the mid-IR range (4000–400 cm^−1^) and nujol mull on a polyethylene plate for the far-IR range (600–100 cm^−1^). Raman spectrum of powdered crystals was measured using a Bruker FT100/S spectrometer (Billerica, MA, USA) with YAG:Nd laser excitation (1064 nm). Polarized and temperature-dependent Raman spectra were measured using a Renishaw InVia Raman spectrometer (Wotton-under-Edge, UK), equipped with confocal DM2500 Leica optical microscope, a thermoelectrically cooled CCD as a detector, and an Ar^+^ ion laser operating at 488 nm. Spectral resolution of both Raman and IR spectra was set to be 2 cm^-1^ and temperature was controlled using Linkam THMS600 cryostat cell (Tadworth, UK), equipped with quartz windows.

### 2.3. Computational Details

**Figure 1:**
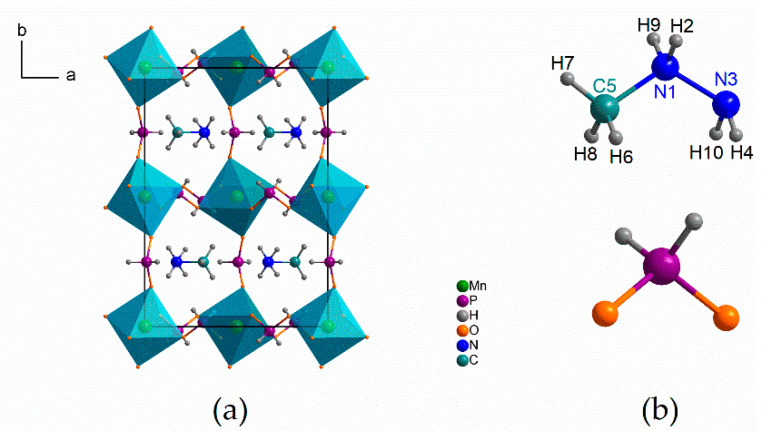
The crystal structure packing of [MHy][Mn(H_2_POO)_3_] taken from Ref. [5] (**a**) and the structural model of MHy^+^ cation (with the atomic numbering used in the Density Functional Theory (DFT) calculations) and H_2_POO^−^ anion (**b**).

The geometry optimization of the molecular structure of the MHy^+^ cation (Figure 1) was performed with the use of Gaussian 03 program package [21]. All calculations were performed using density functional three-parameters hybrid (B3LYP) methods [22,23,24] with the 6-311G(2d,2p) [25,26] basis set. In the geometry optimization, the structural parameters from X-ray diffraction studies were taken as input data. The comparison of the calculated and experimental bond lengths and bond angles is presented in Table S1. Geometry optimization was followed by the calculations of vibrational frequencies. The calculated harmonic frequencies were scaled using scaling factors (0.96 and 0.98) to correct the evaluated wavenumbers for vibrational anharmonicity and deficiencies inherent to the used computational level. Anharmonic wavenumbers were also calculated using deperturbed vibrational second-order perturbation theory (DVPT2). To assign the calculated modes, the Chemcraft computer program was used [27]. The Potential Energy Distribution (PED) of the normal modes among the respective internal coordinates was calculated for studied compound using the BALGA [28] program.

## 3. Results and Discussion

### 3.1. Structure

As follows from Table S1, the calculated C-N and N-N bond lengths (1.506 and 1.447Å, respectively) and C-N-N angle (118.3°) are in very good agreement with the experimental values (1.470, 1.442 Å and 116.3°, respectively [5]). The calculated C-H and N-H bonds are significantly longer than the experimental ones but it should be remembered that X-ray diffraction method is not sensitive for light atoms like hydrogen. The majority of the remaining calculated angles are also in agreement with the experimental ones.

### 3.2. Vibrational Properties

**Figure 2:**
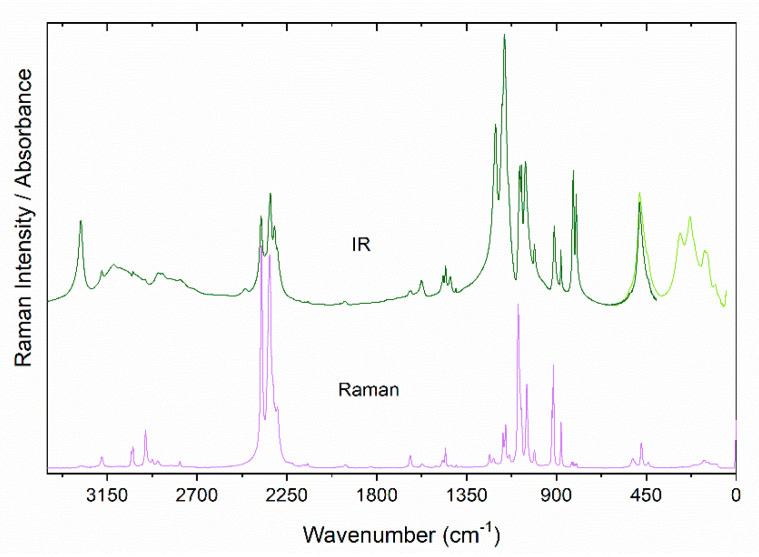
The polycrystalline IR and Raman spectra of the [MHy][Mn(H_2_POO)_3_] perovskite measured at room temperature.

**Figure 3:**
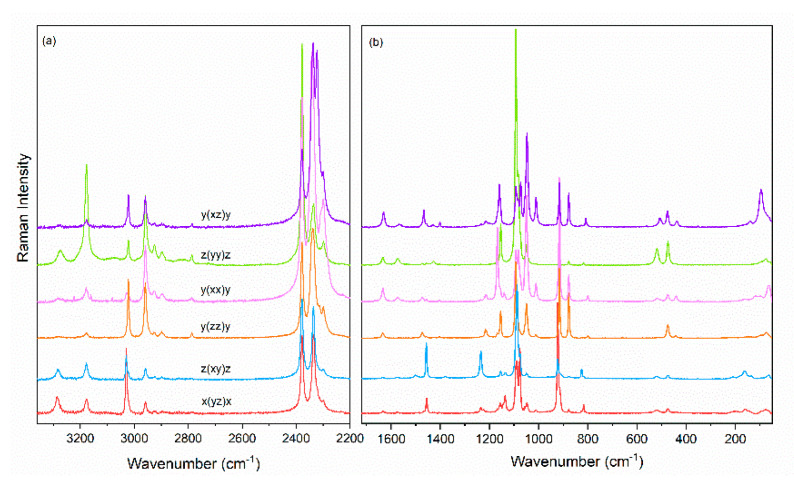
Polarized Raman spectra of the [MHy][Mn(H_2_POO)^3^] single crystal in the (**a**) 2200–3360 cm^−1^ and (**b**) 50–1720 cm^−1^ range.

Experimental IR and Raman spectra of powdered crystals are presented in Figure 2, while polarized Raman spectra are depicted in Figure 3. Table 1 and Table S2 list the calculated and observed experimental wavenumbers as well as the proposed assignment.

**Internal vibrations of MHy^+^ cation**. Free MHy^+^ cation has 24 vibrational degrees of freedom that can be subdivided into symmetric stretching (2ν_s_NH_2_), antisymmetric stretching (2ν_as_NH_2_), scissoring (2δNH_2_), rocking (2ρNH_2_), wagging (2ωNH_2_), and torsion or twisting (2τNH_2_) modes of the NH_2_ and NH_2_^+^ groups; symmetric stretching (ν_s_CNN), antisymmetric stretching (ν_as_CNN) and bending (δCNN) modes of the CNN skeletal group, and symmetric stretching (ν_s_CH_3_), antisymmetric stretching (2ν_as_CH_3_), symmetric bending (δ_s_CH_3_), antisymmetric bending (2δ_as_CH_3_), rocking (2ρCH_3_) and torsion (τCH_3_) modes of the methyl group.

DFT calculations show that the N-H stretching modes of the terminal NH_2_ (middle NH_2_^+^) groups are expected at 3388 + 3306 cm^−1^ (3292 + 3289 cm^−1^) (Table 1). The former modes are observed in the experimental spectra as relatively narrow bands shifted to lower wavenumbers by 30–107 cm^−1^, compared to the calculated ones (Table 1 and Table S2). Small bandwidths and shifts are consistent with involvement of these NH_2_ groups in formation of weak HBs (according to the X-ray diffraction, the N^…^O distance is 3.001 Å [5]). It is worth noting that the 3281 cm^−1^ band is significantly weaker in the Raman spectrum than that observed at 3176 cm^−1^, but opposite behavior is observed in the IR spectrum (Figure 2). Therefore, we assign the 3281 and 3176 cm^−1^ bands to antisymmetric and symmetric stretching, respectively. The modes calculated at 3292 + 3289 cm^−1^ are observed as broad and medium intensity IR bands at 3118 + 3085 cm^−1^ (Figure 2, Table S2). This behavior confirms involvement of the NH_2_^+^ groups in formation of medium strong HBs (the shortest N^…^O distance is 2.875 Å [5]).

The C-H stretching modes of the methyl group are observed as the strongest Raman bands in the 3300–2700 cm^−1^ range. These bands are narrow and those at 3028 and 3021 cm^−1^ have significantly smaller intensity than that at 2958 cm^−1^, allowing their assignment to antisymmetric and symmetric modes, respectively. Raman and IR spectra show also a few bands in the 2930–2700 cm^−1^ range that are not predicted by DFT calculations (Table 1 and Table S2). These bands correspond, therefore, to some combination modes.

The calculated scissoring modes of the NH_2_ and NH_2_^+^ groups (1679 and 1607 cm^−1^, respectively) are in good agreement with the observed ones (1632 and 1574 cm^−1^, Table S2). Good agreement is also observed for the C-H bending modes that are predicted (observed) in the 1472–1439 cm^−1^ (1474–1428 cm^−1^) range. 

According to the DFT calculations, τNH_2_^+^, ωNH_2_, ωNH_2_^+^, ρNH_2_ and ρCH_3_ modes should be observed in the 1449–1031 cm^−1^ range. The mode calculated at 1031 cm^−1^ cannot be observed in our spectra due to overlapping with strong bands of the hypophosphite ion. The remaining modes are, however, observed in the 1403–1136 cm^−1^ range (Table S2). 

In the 1020–770 cm^−1^ range, three bands can be identified as arising from vibrations of MHy^+^ cation. The most intense Raman band at 878 cm^−1^ can be unambiguously assigned to the ν_s_CNN vibration calculated at 811 cm^−1^. DFT predicts that the ρNH_2_ mode should be observed at slightly higher wavenumber (821 cm^−1^) than the ν_s_CNN one. We locate, therefore, this mode in the 915–910 cm^−1^ range. The band observed at 1010 cm^−1^ (calculated at 907 cm^−1^) can be assigned to the ν_as_CNN mode. 

DFT calculations predict the presence of three internal modes of MHy^+^ in the region below 500 cm^−1^. We could identify two of these modes around 440 and 210 cm^−1^ and assign them to δCNN and τCH_3_, respectively (Table 1 and Table S2).

Unit cell of [MHy][Mn(H_2_PO_2_)_3_] contains four MHy^+^ cations located at sites of C_s_ symmetry. Therefore, all internal modes of this cation should split into four components (see Table S3). Raman spectra clearly show that many A_g_ modes are observed at slightly different wavenumbers than their B_1g_, B_2g_ or B_3g_ counterparts, i.e., the significant Davydov splitting is observed (Table S2). The largest differences are observed for the δNH_2_^+^ mode (1574 and 1568 cm^−1^ for the A_g_ and B_2g_ mode, respectively) and δ_as_CH_3_ mode (1451/1452 cm^−1^ and 1457 cm^−1^ for the A_g_ and B_1g_ mode, respectively). In other cases, the differences between wavenumbers of Raman components are less than 1–3 cm^−1^. For instance, the symmetric stretching mode of the CH_3_ group (ν_s_CH_3_) is visible at 2959 cm^−1^ for A_g_ and B_2g_ modes and at 2958 cm^−1^ for B_1g_ and B_3g_ modes. Similar slight changes in the positions of Davydov components are observed for its antisymmetric counterpart (ν_as_CH_3_), at 2959 cm^−1^ for A_g_ and B_2g_ modes and at 2958 cm^−1^ for B_1g_ and B_3g_ modes. It is worth mentioning that the size of Davydov splitting depends on the strength of intermolecular interactions occurring in the studied compound, namely, stronger interactions lead to larger Davydov splitting. 

It is worth making a short comparison of the MHy^+^ modes observed in this hypophosphite and related formate ([MHy][Mn(HCOO)_3_]) reported in the literature. According to the X-ray diffraction data, MHy^+^ cations are ordered in the hypophosphite and the terminal NH_2_ groups are involved in weak HBs, whereas in the formate these cations are disordered and the terminal groups form no HBs [5,8]. Furthermore, MHy^+^ cation shifts away from the cavity center in the hypophosphite but sits in the center in the formate. This difference is related to more flexible P-O-Mn bindings compared to the C-O-Mn ones [5]. Raman and IR spectra reflect these differences very clearly. Firstly, stretching modes of the terminal NH_2_ groups are shifted to higher wavenumbers for the formate (3317 and 3195 cm^−1^ [8]) due to lack of HBs interactions. The skeletal modes are, however, observed at very similar wavenumbers for the both analogues (the largest difference is only 3 cm^−1^), reflecting similar geometry of MHy^+^ cations. Secondly, stretching modes of the methyl groups in the formate are shifted up to 9 cm^−1^ due to larger constraint imposed on the MHy^+^ cations. Thirdly, IR band corresponding to the ρNH_2_^+^ mode is very broad for the formate (about 30 cm^−1^ at RT) compared to the hypophosphite (12.2 cm^−1^), reflecting disorder in the former case.

**Internal vibrations of hypophosphite anion**. The free hypophosphite ion has C_2v_ symmetry and 9 fundamental vibrations, i.e., symmetric stretching (ν_s_(PH_2_), A_1_), antisymmetric stretching (ν_as_(PH_2_), B_1_), scissoring (δ(PH_2_), A_1_), rocking (ρ(PH_2_), B_1_), twisting (τ(PH_2_), A_2_) and wagging (ω(PH_2_), B_2_) modes of the PH_2_ groups as well as symmetric stretching (ν_s_(PO_2_), A_1_), antisymmetric stretching (ν_as_(PO_2_), B_2_) and scissoring (δ(PO_2_), A_1_) modes of the PO_2_ moiety [29].

Former experimental and theoretical studies showed that five of the mentioned above vibrations of H_2_PO_2_^−^ anion are observed in well-separated regions. That is, ν_s_(PH_2_) at 2345–2424 cm^−1^, ν_as_(PH_2_) at 2305–2368 cm^−1^, τ(PH_2_) at 899–945 cm^−1^, ρ(PH_2_) at 792–834 cm^−1^ and δ(PO_2_) at 456–518 cm^−1^ [20,30,31,32]. For [MHy][Mn(H_2_PO_2_)], these modes are observed at 2333–2379, 2297–2322, 910–921, 799–824 and 474–519 cm^−1^, respectively (Table S2). 

According to the literature data, the ν_as_(PO_2_) and δ(PH_2_) modes are observed in a similar wavenumber range, typically at 1154–1230 and 1132–1172 cm^−1^, respectively [20,30,31,32]. The same behavior is observed for the ν_s_(PO_2_) and ω(PH_2_) modes that are usually observed at 1024–1062 and 1070–1126 cm^−1^, respectively [30,31,32]. Our Raman and IR spectra of [MHy][Mn(H_2_PO_2_)] show two separated groups of bands at 1154–1204 and 1048–1092 cm^−1^. The former bands can be assigned to ν_as_(PO_2_) and δ(PH_2_) modes and the latter to ν_s_(PO_2_) and ω(PH_2_) ones. 

It is worth adding that there are two types of H_2_PO_2_^−^ ions in the [MHy][Mn(H_2_PO_2_)] structure, one located at site of C_s_ and second at site of C_1_ symmetry [5]. Each fundamental mode of the former (later) anion should split into 4 (8) Davydov components (see Table S3). Indeed, Raman and IR spectra clearly show splitting of each mode into a few bands (Table S2). For instance, two τ(PH_2_) modes are observed due to two crystallographically distinct H_2_PO_2_^−^ ions. Wavenumbers of these modes practically do not depend on polarization, pointing to negligible Davydov splitting. However, the ν_s_(PH_2_), and ν_as_(PH_2_) and δ(PO_2_) split into three components and the ρ(PH_2_) into four components, indicating significant Davydov splitting for these modes.

**Lattice modes.** Mn^2+^ ions are located in the structure at inversion center and as a result, T’(Mn^2+^) mode, are IR-active only (see Table S3). Our IR spectrum shows two strong IR bands at 229 and 280 cm^−1^ that have no Raman counterparts. These bands can be unambiguously assigned to T’(Mn^2+^) modes. It is worth noting here that this assignment is consistent with studies of zinc hypophosphites, which revealed T’(Zn^2+^) modes at 234–357 cm^−1^ [32]. 

Modes in the 137-162 cm^−1^ range are much stronger in the IR than Raman spectra while opposite behavior can be noticed for the modes located below 120 cm^−1^. This behavior is consistent with assignment of the former bands to the coupled modes involving translational motions of T’(Mn^2+^) and T’(H_2_PO_2_^−^) ions and the latter ones to librational L(H_2_PO_2_^−^) modes.

### 3.3. Temperature Dependence of Raman Modes

**Figure 4:**
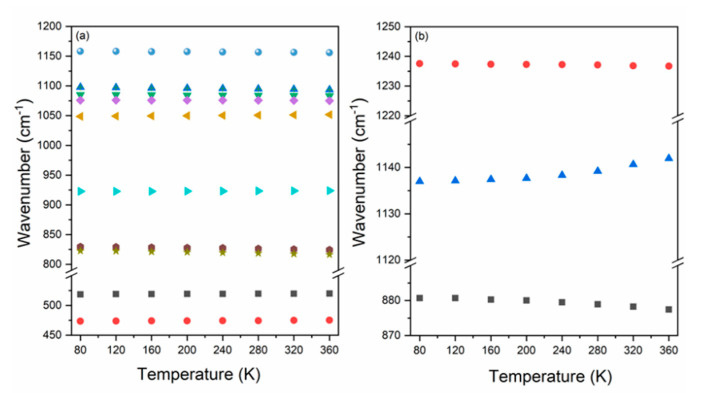
The temperature evolution of Raman wavenumbers of bands corresponding to vibrations of groups that built up (**a**) H_2_PO_2_^−^ and (**b**) MHy^+^ ions.

**Figure 5:**
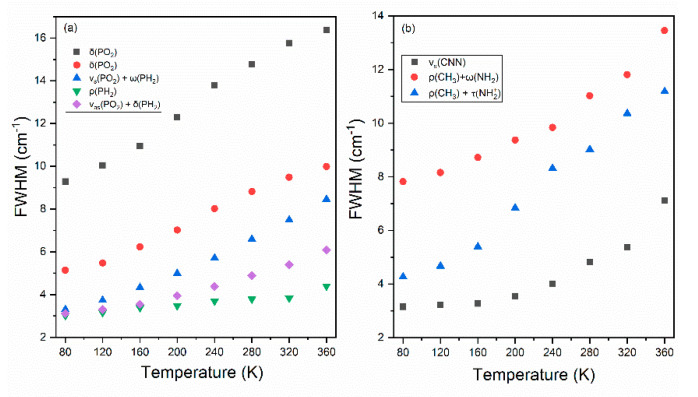
The temperature evolution of Raman full widths at half maximum (FWHMs) of bands corresponding to vibrations of groups that built up (**a**) H_2_PO_2_^−^ and (**b**) MHy^+^ ions.

Raman spectra of the [MHy][Mn(H_2_PO_2_)] sample recorded as a function of temperature (80–360 K) are presented in Figure S1. Increasing of temperature leads to broadening and shift of bands. As a consequence, many bands are overlapped and hardly resolved above the room temperature. Since splitting as well as an emergence of additional or a disappearance of already present bands is not observed, the occurrence of a structural phase transition in [MHy][Mn(H_2_PO_2_)] can be excluded, in agreement with the literature [5]. This conclusion is further supported by the temperature evolution of wavenumbers and full width at half maximum (FWHM) values for a few selected Raman modes (Figure 4 and Figure 5), which do not show any splitting or departures from the typical anharmonic behavior in the studied temperature range.

Figure 4 presents the temperature-dependent evolution of bands assigned to vibrations of PH_2_ and PO_2_ groups building H_2_PO_2_^−^ liners. As can be seen, Raman bands exhibit weak hardening upon cooling from 360 K to 80 K, that is, the observed shifts do not exceed 6 cm^−1^. The largest shift concerns bands located at 823 and 830 cm^−1^ (360 K) attributed to rocking motions of PH_2_ groups (ρPH_2_), which upon cooling move toward lowest wavenumbers by about 6 cm^−1^. Much weaker changes, i.e., upshifts by 1 or 2 cm^−1^, can be observed for bands assigned to bending motions of PO_2_ groups (δPO_2_) located at 519 and 473 cm^−1^ (at 360 K). Interesting observations can also be made for temperature dependence of FWHM of bands corresponding to vibrations of PH_2_ and PO_2_ groups (Figure S5a). In this case, modes narrow significantly upon cooling. For instance, the bandwidth of 519 cm^−1^ (RT) Raman band (δPO_2_) decreases from 16.4 cm^−1^ (360 K) to 9.3 cm^−1^ (80 K). Much weaker narrowing from 4.4 cm^−1^ (360 K) to 3.0 cm^−1^ (80 K) applies to band located at 822 cm^−1^ (RT), which has the main contribution of the rocking vibration of PH_2_ groups. Differences in thermal behavior for different vibrational modes of PH_2_ and PO_2_ groups can be explained by various surrounding and their different ability to form H-bonding. Please note that two inequivalent H_2_PO_2_^−^ units coordinate neighboring Mn^2+^ ions in a different way, and thus the created P-O-Mn angles vary in a broad range (127, 130 and 157°) [5]. These linkers are also involved in the formation of hydrogen bonds (HBs) with the NH_2_ groups from MHy^+^ cations, which is directly responsible for the off-center displacement of organic ions inside the cavities. Decrease of temperature leads to slowing down of thermally-activated motions followed by decrease of the unit cell volume and increase of the HBs strength. Since the impact of HBs is not the same for the PH_2_ and PO_2_ groups, different thermal behavior for modes attributed to their vibrations can be observed.

Similar behavior also applies to bands assigned to vibrational motions of MHy^+^ cations (Figure 4b). In this case, the observed shifts do not exceed 5 cm^−1^. The largest changes are observed for bands at 1142 cm^−1^ and 881 cm^−1^ (360 K). Upon cooling, the first peak corresponding to mixed rocking and torsion vibrations of the NH_2_ groups exhibits downshift to 1137 cm^−1^, whereas the second one originating from symmetric stretching vibrations of the CNN moieties changes its position to 877 cm^−1^. Meanwhile, minimal hardening of 0.8 cm^−1^ upon cooling is noticed for the band at 1237 cm^−1^ (RT) attributed to mixed rocking vibrations of the CH_3_ groups and wagging motions of the NH_2_ groups. As in the case of vibrations of H_2_PO_2_^−^ linkers, observed differences in thermal evolution of modes attributed to vibrational motions of MHy^+^ ions can also be explained by different influence of HBs on particular groups in the structure. 

The thermal behavior of FHWM of the 878 cm^−1^ (RT), 1237 cm^−1^ and 1139 cm^−1^ bands corresponding to the ν_s_CNN, ρCH_3_+ωNH_2_ and ρCH_3_+τNH_2_^+^ modes, respectively, is presented at Figure 5b. As can be seen, upon cooling all mentioned bands exhibit significant narrowing. The larger narrowing, by almost 9 cm^−1^, is observed for the band attributed to mixed rocking vibration of the CH_3_ groups and twisting vibrations of the NH_2_^+^ groups. Observed results prove that change of temperature leads to strong change in the HB strengths.

## 4. Conclusions

Phonon properties of manganese hypophosphite framework templated by methylhydrazinium cations were studied using IR and Raman spectroscopies. We have presented selection rules for the *Pnma* orthorhombic phase together with proposed assignment of the observed IR and Raman bands to the respective internal and lattice vibrations. Presented ascription was based on our DFT calculations made for the isolated MHy^+^ ion in harmonic and anharmonic approximations. Polarized Raman spectra provided additional information on magnitude of Davydov splitting observed in the studied organic-inorganic hybrid. The temperature-dependent Raman studies allowed us to obtain deeper insight into structural changes occurring in the [MHy][Mn(H_2_POO)_3_] compound. Differences in the temperature evolution of wavenumbers and FWHMs of bands attributed to vibrational motions of the MHy^+^ as well as H_2_PO_2_^−^ ions can be explained by the different influence of HB on individual groups that built this structure.

## Figures and Tables

**Table 1:** Calculated (harmonic and anharmonic) wavenumbers for the MHy^+^ cation.

No.	ν_harm_	ν_harm_^1^	ν_anharm_	PED
1	3564	3421	3388	ν_as_NH_2_-99
2	3477	3338	3306	ν_s_NH_2_-100
3	3461	3323	3292	ν_as_NH_2_^+^-99
4	3425	3288	3289	ν_s_NH_2_^+^-98
5	3180	3053	3049	ν_as_CH_3_-100
6	3166	3039	3029	ν_as_CH_3_-100
7	3075	2952	3014	ν_s_CH_3_-100
8	1700	1666	1679	δNH_2_-80 + δNH_2_^+^-20
9	1670	1637	1607	δNH_2_^+^-80 + δNH_2_-20
10	1499	1469	1472	δ_as_CH_3_-91
11	1499	1469	1458	δ_as_CH_3_-62 + ρNH_2_-22 + τNH_2_^+^-14
12	1490	1460	1449	τNH_2_^+^-38 + δ_as_CH_3_-35 + ρNH_2_-26
13	1461	1432	1439	δ_s_CH_3_-97
14	1443	1415	1401	ωNH_2_^+^-86 + δ_as_CH_3_-15
15	1336	1309	1297	τNH_2_^+^-41 + ρCH_3_-36 + ρNH_2_-22
16	1219	1194	1176	ρCH_3_-50 + ωNH_2_-25 + νNH_2_^+^-NH_2_-13 + δNH_2_^+^-13
17	1117	1095	1091	ρCH_3_-42 + ρNH_2_-25 + τNH_2_^+^-32
18	1065	1043	1031	ωNH_2_-37 + ρCH_3_-30 + νNH_2_^+^-NH_2_-20 + νNH_2_^+^-CH_3_-12
19	953	934	907	νNH_2_^+^-CH_3_-48 + ωNH_2_-27 + νNH_2_^+^-NH_2_-22
20	841	824	821	ρNH_2_^+^-75 + ρCH_3_-22
21	836	819	811	νNH_2_^+^-NH_2_-46 + νNH_2_^+^-CH_3_-37 + ωNH_2_-12
22	405	397	404	δCNN-87
23	281	275	252	τNH_2_-73 + τCH_3_-27
24	225	220	245	τCH_3_-73 + τNH_2_-27

^1^ scaling factor = 0.98 (2499–0 cm^−1^) + 0.96 (3500–2500 cm^−1^); abbreviations: in-plane vibrations: ν—stretching; δ*—*bending; ρ—rocking; out-of-plane vibrations: ω—wagging; τ—twisting

## Supplementary Materials

The following are available online, Figure S1: The temperature-dependent Raman spectra, Table S1: Selected bond lengths and bond angles of MHy^+^ cation. Table S2: Raman and IR wavenumbers of [MHy][Mn(H_2_POO)_3_] at RT, Table S3: The correlation diagram and irreducible representations for the *Pnma* orthorhombic phase of the [MHy][Mn(H_2_POO)_3_] crystal.
