# Supplementary material for: Vibrational Properties and DFT Calculations of Perovskite-Type Methylhydrazinium Manganese Hypophosphite
Source: Molecules. 2020 Nov 9;25(21):5215. doi: 10.3390/molecules25215215 (PMC7664875; doi:10.3390/molecules25215215)
Supplement: Supplementary file 1 [file molecules-25-05215-s001.pdf]

Vibrational properties and DFT calculations of perovskite-type methylhydrazinium manganese hypophosphite

Aneta Ciupa-Litwa, Maciej Ptak*, Edyta Kucharska, Jerzy Hanuza, Mirosław Mączka

* m.ptak@intibs.pl

Table S1. Selected bond lengths (Å) and bond angles (°) of MHy^+ cation.

Bond lengths and angles	Calculated	Observed	Bond lengths and angles	Calculated	Observed
C5–H6	1.086	0.9596	N1–H9	1.023	0.8902
C5–H7	1.088	0.9596	N1–N3	1.447	1.4415
C5–H8	1.086	0.9596	N1–H4	1.016	0.8659
C5–N1	1.506	1.4698	N3–H10	1.017	0.8659
N1–H2	1.023	0.8902			
C5–N1–N3	118.30	116.371	H4–N3–H10	108.05	95.230
N1–C5–H6	110.56	109.458	N3–N1–H2	106.10	108.218
N1–C5–H7	108.39	109.525	N3–N1–H9	106.09	108.218
N1–C5–H8	108.24	109.456	N1–N3–H4	107.62	109.988
C5–N1–H2	110.17	108.189	N1–N3–H10	107.62	109.988
C5–N1–H9	110.17	108.189			
H2–N1–H9	105.14	107.318			

Table S2. Raman and IR wavenumbers of $[MHy][Mn(H_2POO)_3]$ at RT. [1]

Polycrystalline		Polarized Raman						
Raman	IR	y(xx)y A_g	z(yy)z A_g	y(zz)y A_g	y(xz)y B_{2g}	z(xy)z B_{1g}	x(yz)x B_{3g}	Assignment
3281w	3281s	3279vww	3274m		3283vw	3283m	3286m	$\nu_{as}NH_2$
3176w	3176w	3177w	3178s	3177w	3179vw	3177m	3176m	$\nu_{s}NH_2$
	3118m,b							νNH_2^+
	3085m,b		3077w,b					νNH_2^+
3028m		3028w				3030m	3029m	$\nu_{as}CH_3$
3021m	3020w	3022w	3023w	3022m	3020m	3021sh	3020sh	$\nu_{as}CH_3$
2958m	2958vw	2959m	2959m	2959m	2959m	2958m	2958m	$\nu_{s}CH_3$
2924w		2926w	2924w	2924vw	2927vw	2928vw		combination
2898w	2894w	2894w	2897w	2897vw	2892vw	2897vw		combination
	2874w							combination
	2834w		2828w,b					combination
2785w	2783w	2783vw	2785w	2785vw	2784vw			combination
2377vs	2379s	2378s	2378s	2378s	2377m	2378m	2378m	$\nu_{s}PH_2$
2342sh	2341sh	2342s	2342sh	2340s				$\nu_{s}PH_2$
2336vs	2333s	2336sh	2335m	2335s	2338s	2336m	2335m	$\nu_{s}PH_2$
2322w					2322s		2322sh	$\nu_{as}PH_2$
2311ww	2311m	2309m		2311sh				$\nu_{as}PH_2$
2297w	2297w	2299m	2299w	2299w	2299vw	2297w	2300w	$\nu_{as}PH_2$
1632w	1636w	1633w	1633vw	1633vw	1630w	1632vw	1632vw	δNH_2
	1629w							δNH_2
1574w	1575m	1574vw	1574vw		1568vw	1572vw	1572vw	δNH_2^+
1473w	1474sh	1474w	1472vw	1472w				$\delta_{as}CH_3$
1466w	1467w				1466w			$\delta_{as}CH_3$
1456w	1455w	1451vw	1452vw	1452vw	1455sh	1457m	1456m	$\delta_{as}CH_3$
1428vw	1431w		1428vw		1427vw	1422vw		$\delta_{s}CH_3$
1402vw	1403w	1401vw	1403vw	1403vw	1402vw			τNH_2^+
1377vw						1380w		ωNH_2^+
1237w					1234vw	1235m	1235w	$\rho CH_3+\omega NH_2$
1214w	1215sh	1215vw		1215w	1215vw		1220w	$\rho CH_3+\omega NH_2$
	1205s							$\nu_{as}PO_2+\delta PH_2$
1169w	1171w	1168m	1168w	1168w	1168sh		1166sh	$\nu_{as}PO_2+\delta PH_2$
1157sh	1160s	1159sh			1160m			$\nu_{as}PO_2 + \delta PH_2$
1154w			1155m	1155m		1155w	1156w	$\nu_{as}PO_2 + \delta PH_2$
1139w	1138sh	1140w				1136w	1136m	$\rho CH_3 + \tau NH_2^+$
1095s		1092m	1092s	1092s	1092m	1092sh	1091s	$\nu_{s}PO_2 + \omega PH_2$

1088sh						1088s	1088s	$\nu_s PO_2 + \omega PH_2$
1083sh	1083m	1082m	1084sh	1084sh	1085m			$\nu_s PO_2 + \omega PH_2$
1075w	1075m	1074sh	1074sh	1074sh	1072m	1076sh	1076s	$\nu_s PO_2 + \omega PH_2$
		1056sh		1055sh	1056sh		1057w	$\nu_s PO_2$
1050m	1054m	1050m	1050w	1050m	1048m	1049w	1048w	$\nu_s PO_2$
1010w	1010w	1011w		1011vw	1011m		1011w	$\nu_{as} CNN$
923m					921sh	921m	921s	τPH_2
915m	910m	916s		916s	916m	915sh	915sh	$\tau PH_2 + \rho NH_2^+$
878m	877m	877m	877vw	877s	877m	878vw	877w	$\nu_s CNN$
826w						824w	824vw	ϱPH_2
818w	818s		817vw			817vw	816w	ϱPH_2
	815s							ϱPH_2
807w	805sh				808vw			ϱPH_2
799w	801s	800w		800vw				ϱPH_2
519w		518vw	518w			518w	518w	δPO_2
506sh					505w			δPO_2
474w	483s	474w	474m	474m	476w	474w	474w	δPO_2
441w	440sh	441w		441vw	438w			δCNN
	280s							T′(Mn^{2+})
	229s							T′(Mn^{2+})
210w	213sh					207w	201w,b	τCH_3
160w	158m	159w				161w	160w	T′(Mn^{2+}) +T′(H_2POO^-)
141w	146m			146vw,b	142vw	135w	142w	T′(Mn^{2+})+T′(H_2POO^-) +T′(MHy^+)
118w		118w						L(H_2POO^-)
95w	104vw	94w	94vw	94vw	95m		95w	L(H_2POO^-)
75w			75w	74w	70w,b		77w	L(H_2POO^-)
64m		64m	61vw			65w	61w	L(H_2POO^-)

[1] key: vs, very strong; s, strong; m, medium; w, weak; vw, very weak; b, broad

Table S3. The correlation diagram and irreducible representations (Γ) for the *Pnma* orthorhombic phase of the [MHy][Mn(H_2POO)$_3$] crystal. [2]

Ion	Vibration	Free ion symmetry	Site symmetry	Factor group symmetry
		***C**$_s$*	***C**$_s$*	***D**$_{2h}$*
	$\nu_s NH_2$ and $\nu_s NH_2^+$	2A′	2A′	$2A_g+2B_{2g}+2B_{1u}+2B_{3u}$
	$\nu_{as} NH_2$ and $\nu_{as} NH_2^+$	2A″	2A″	$2B_{1g}+2B_{3g}+2A_u+2B_{2u}$
	δNH_2 and δNH_2^+	2A′	2A′	$2A_g+2B_{2g}+2B_{1u}+2B_{3u}$
	ωNH_2 and ωNH_2^+	2A′	2A′	$2A_g+2B_{2g}+2B_{1u}+2B_{3u}$
	τNH_2 and τNH_2^+	2A″	2A″	$2B_{1g}+2B_{3g}+2A_u+2B_{2u}$
	ϱNH_2 and ϱNH_2^+	2A″	2A″	$2B_{1g}+2B_{3g}+2A_u+2B_{2u}$
	ν_{as}CNN	A′	A′	$A_g+B_{2g}+B_{1u}+B_{3u}$
	ν_sCNN	A′	A′	$A_g+B_{2g}+B_{1u}+B_{3u}$
MHy^+	δCNN	A′	A′	$A_g+B_{2g}+B_{1u}+B_{3u}$
	$\nu_s CH_3$	A′	A′	$A_g+B_{2g}+B_{1u}+B_{3u}$
	$\nu_{as} CH_3$	A′+A″	A′+A″	$A_g+B_{1g}+B_{2g}+B_{3g}+A_u+B_{1u}+B_{2u}+B_{3u}$
	$\delta_s CH_3$	A′	A′	$A_g+B_{2g}+B_{1u}+B_{3u}$
	$\delta_{as} CH_3$	A′+A″	A′+A″	$A_g+B_{1g}+B_{2g}+B_{3g}+A_u+B_{1u}+B_{2u}+B_{3u}$
	ϱCH_3	A′+A″	2A′	$A_g+B_{1g}+B_{2g}+B_{3g}+A_u+B_{1u}+B_{2u}+B_{3u}$
	τCH_3	A″	A″	$B_{1g}+B_{3g}+A_u+B_{2u}$
	T′	2A′+A″	2A′+A″	$2A_g+B_{1g}+2B_{2g}+B_{3g}+A_u+2B_{1u}+B_{2u}+2B_{3u}$
	L	A′+2A″	A′+2A″	$A_g+2B_{1g}+B_{2g}+2B_{3g}+2A_u+B_{1u}+2B_{2u}+B_{3u}$
	Γ	17A′+13A″	17A′+13A″	$17A_g+13B_{1g}+17B_{2g}+13B_{3g}+13A_u+17B_{1u}+13B_{2u}+17B_{3u}$
		***C**$_{2v}$*	***C**$_s$ (**C**$_1$)*	***D**$_{2h}$ (**D**$_{2h}$)*
	$\nu_s(PH_2)$	A_1	A′ (A)	$A_g+B_{2g}+B_{1u}+B_{3u}$ ($A_g+B_{1g}+B_{2g}+B_{3g}+A_u+B_{1u}+ B_{2u}+B_{3u}$)
	$\nu_{as}(PH_2)$	B_1	A′ (A)	$A_g+B_{2g}+B_{1u}+B_{3u}$ ($A_g+B_{1g}+B_{2g}+B_{3g}+A_u+B_{1u}+ B_{2u}+B_{3u}$)
	$\nu_s(PO_2)$	A_1	A′ (A)	$A_g+B_{2g}+B_{1u}+B_{3u}$ ($A_g+B_{1g}+B_{2g}+B_{3g}+A_u+B_{1u}+ B_{2u}+B_{3u}$)
	$\nu_{as}(PO_2)$	B_2	A″ (A)	$B_{1g}+B_{3g}+A_u+B_{2u}$ ($A_g+B_{1g}+B_{2g}+B_{3g}+A_u+B_{1u}+ B_{2u}+B_{3u}$)
	$\varrho(PH_2)$	B_1	A′ (A)	$A_g+B_{2g}+B_{1u}+B_{3u}$ ($A_g+B_{1g}+B_{2g}+B_{3g}+A_u+B_{1u}+ B_{2u}+B_{3u}$)
	$\tau(PH_2)$	A_2	A″ (A)	$B_{1g}+B_{3g}+A_u+B_{2u}$ ($A_g+B_{1g}+B_{2g}+B_{3g}+A_u+B_{1u}+ B_{2u}+B_{3u}$)
H_2POO^-	$\omega(PH_2)$	B_2	A″ (A)	$B_{1g}+B_{3g}+A_u+B_{2u}$ ($A_g+B_{1g}+B_{2g}+B_{3g}+A_u+B_{1u}+ B_{2u}+B_{3u}$)
	$\delta(PH_2)$	A_1	A′ (A)	$A_g+B_{2g}+B_{1u}+B_{3u}$ ($A_g+B_{1g}+B_{2g}+B_{3g}+A_u+B_{1u}+ B_{2u}+B_{3u}$)
	$\delta(PO_2)$	A_1	A′ (A)	$A_g+B_{2g}+B_{1u}+B_{3u}$ ($A_g+B_{1g}+B_{2g}+B_{3g}+A_u+B_{1u}+ B_{2u}+B_{3u}$)
	T′	$A_1+B_1+B_2$	2A′+ A″ (3A)	$2A_g+B_{1g}+2B_{2g}+B_{3g}+A_u+2B_{1u}+B_{2u}+2B_{3u}$ ($3A_g+3B_{1g}+3B_{2g}+3B_{3g}+3A_u+3B_{1u}+3B_{2u}+3B_{3u}$)
	L	$A_2+B_1+B_2$	A′+ 2A″ (3A)	$A_g+2B_{1g}+B_{2g}+2B_{3g}+2A_u+B_{1u}+2B_{2u}+B_{3u}$ ($3A_g+3B_{1g}+3B_{2g}+3B_{3g}+3A_u+3B_{1u}+3 B_{2u}+3B_{3u}$)
	Γ	$5A_1+2A_2+4B_1+4B_2$	9A′+ 6A″ (15A)	$9A_g+6B_{1g}+9B_{2g}+6B_{3g}+6A_u+9B_{1u}+6B_{2u}+9B_{3u}$ ($15A_g+15B_{1g}+15B_{2g}+15B_{3g}+15A_u+15B_{1u}+15 B_{2u}+15B_{3u}$)
		***C**$_i$*		***D**$_{2h}$*
Mn^{2+}	T		$3A_u$	$3A_u+3B_{1u}+ 3B_{2u}+3B_{3u}$
	Γ		$3A_u$	$3A_u+3B_{1u}+ 3B_{2u}+3B_{3u}$

[2] key: red, IR-active; green, Raman-active; blue, IR- and Raman-active.

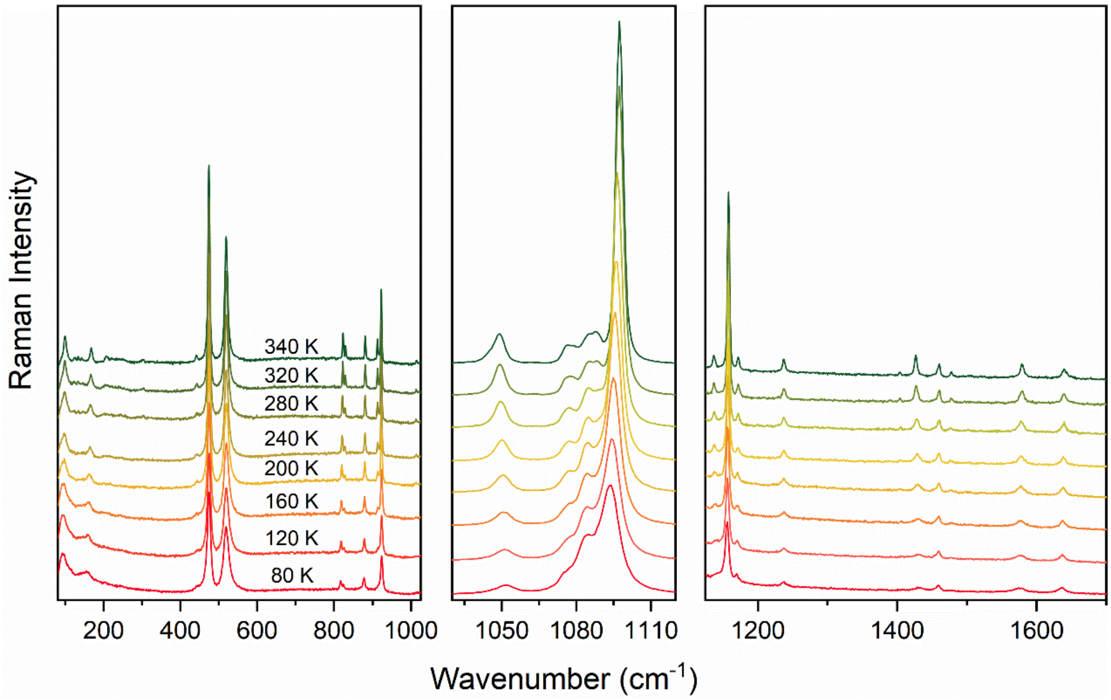

Figure S1. The temperature-dependent Raman spectra of the [MHy][Mn(H_2POO)$_3$] perovskite.
